# Effects of oil spills on fish production in the Niger Delta

**DOI:** 10.1371/journal.pone.0205114

**Published:** 2018-10-25

**Authors:** Eze Simpson Osuagwu, Eseoghene Olaifa

**Affiliations:** Department of Economics and Development Studies, Covenant University, Ota, Nigeria; Havforskningsinstituttet, NORWAY

## Abstract

The Niger Delta region is the oil producing area of Nigeria, which consists of highly diverse ecosystems that are supportive of numerous species of terrestrial and aquatic fauna and flora. Crude oil spills endanger fish hatcheries in coastal water and also contaminate valuable fish. This study examines the effects of oil spills on fish production in the Niger Delta of Nigeria from 1981–2015 using an estimable Cobb Douglas production function. The findings suggest that oil production and spills negatively affect fish production, while farm labour has a positive effect on fish production. On the other hand, fishery loan exerts a negative effect on fish production and this could be ascribed to the bottlenecks in accessing these loans. This study corroborates the findings in literature on the negative concomitance of oil spills and fish production and suggests a cautious approach to oil exploration activities for a sustainable development in the region.

## Introduction

In the Niger Delta, oil spill is perceived to be a major consequence of an inordinate exploitation of petroleum resources in the region. Some recent studies have examined spatial variations in the natural mortality of particular fish species in order to assess the impact of oil spills on marine ecosystems, specifically in the Arctic area [1, 2 – 3]. But, varying results from these studies indicate the resistance of fish species to the toxicity of the environment. However, this study examines oil spills as a major environmental problem that hampers fish production drawing from econometric techniques of the Cobb-Douglas production function, ignoring spatial variations due to data constraints. Above all, we seek to ascertain a statistical relationship and suggest possible policy implications for a sustainable economic environment for increasing fish production and food sufficiency in one of the most degraded areas in the world. This study differs from previous research on environmental degradation in the Niger Delta and implications on agricultural productivity by specifically examining fish production from the perspective of analysing data on fresh water fishing.

The Niger Delta is the major oil producing region of Nigeria, which is located by the Atlantic Coast where River Niger divides into numerous tributaries. The region is known to be the second largest delta in the world of about 450 kilometres of coast line that terminates at the Imo River entrance [4]. This region spans through 20,000 square kilometres and has been described as the largest wetland in Africa and among the three largest in the world, consisting about 2,370 square kilometres of rivers, creeks and estuaries and stagnant swamp covering about 8,600 square kilometres [5 – 6]. This highly diverse ecosystem supports numerous species of aquatic flora and fauna and terrestrial life, [7] describes the Niger Delta area as the one of the richest wetlands in the world. The exploitation of oil in the Niger Delta region has brought to bear oil spillage and its numerous problems. Such problems include contamination of water bodies, danger to aquatic life, and destruction of farmlands, [8]. According to [8], between 1976 and 1996, it was estimated that over 6,000 oil spills occurred in the Niger Delta region and about 2-million barrels of crude oil leaked into the environment. This calls for serious concern knowing that this ecosystem is a major source of livelihood for the inhabitants of the region.

Oil was first discovered in the region in 1956 and since the early 1970s oil has dominated the country’s economy. Oil exploration has over the years impacted negatively on the physical environment of the oil-bearing communities [9]. [10] observes that oil exploitation has increased the rate of environmental degradation and has perpetuated food insecurity as a result of death of fish and crops as well as loss of farm lands and viable rivers for fishing activities leading to loss of livelihood. There is no doubt that the disastrous effect of oil spill impedes agricultural productivity and fishing to be specific, which in the long-run has an adverse consequence on the economic life of the inhabitants of this region [11, 9]. Furthermore, studying the prospects and challenges of environmental impact of oil exploration in the Niger Delta region of Nigeria and the remediation of contaminated lands in the region, [12] argues that resolving the technical dilemma of the clean-up mechanism and identifying social impediments will be the key success driver of the United Nations Environmental Programme action plan, which was recently adopted by the government of Nigeria for the clean-up of the Niger Delta. The study further recommends that bioremediation should be adopted considering its low greenhouse effect and the reduced cost burden on the weak and overstretched economy of Nigeria. The motivation for this study draws from the increasing importance of fish to the economic life of this region and the country at large, with the attendant negative effect of oil exploration activities. In this study, we provide specific answers to the problem of environmental degradation through oil spills on fish production in the Niger Delta region by applying econometric techniques. Also, the effect of positive actions such as agricultural credit to fishermen is considered as an explanatory variable to reduce omitted variable bias. This study is presented in six sections, the second is a review of related literature, the third is a presentation of stylized facts, while the fourth section discusses the method of analysis, fifth is the interpretation and discussion of results and the study concludes in the sixth section.

## Literature review

Studies on the impact of oil spills on fish production are becoming increasing important, not only because it provides the basis for understanding the reprehensible activities of oil exploration but uncovers the ideological and intellectual perspective of the relationship between oil spills and environmental degradation [13, 14, 15]. [16] examines the environmental impact of oil exploration and exploitation in Niger Delta of Nigeria using tabular analysis of data obtained from secondary sources. The study finds that the oil industry sited within this region has contributed enormously to the economic growth of the country, but unsustainable oil exploration activities have rendered the Niger Delta region one of the five most severely damaged ecosystems in the World. Nevertheless, [17] assessed oil exploration and spillage in the Niger Delta region of the country, using comparative analysis of secondary data covering periods from 1976 to 2000 on descriptive techniques such as line and bar graphs, and found a decrease in oil spillage quantity but an increase in the number and times of oil spill. On the other hand, the findings in [15] provides evidence for the decrease in agricultural yield over the period following persistent environmental degradation due to oil spills and oil pipeline vandalization.

[18] estimates the effects of oil pollution on crop production in Rivers State, Nigeria on a sample of 296 respondents drawn from 17 out of 23 Local Government Areas, applied a stochastic trans-log production function in a multi-stage sampling technique. The results indicate that the effect of crude oil pollution on crop farms reduced the size of farmland, significantly at 1%, reducing marginal physical product (MPP), while in non-polluted farms output increased. Physical inputs, crude oil pollution variables and their interactions show strong negative (diminishing) returns to scale in oil polluted farms, but in non-polluted farmlands result indicate strong positive returns to scale. The technical efficiency results show that less than 22% of crop farmers were over 80% efficient in their use of resources in oil polluted farmlands, while technical efficiency in non-polluted farmlands indicates a high efficiency of 33%. This result indicates that environmental degradation poses a serious threat to farmers by diminishing both physical ability and psychological desires to farm. The goal of farming may be defeated before the proper exercise, especially when the individual has no hope of any compensation when the crops are destroyed, or the waters are polluted, as always, the case in the Niger delta region.

[19] critically assessed the effect of oil exploration on poverty in the Niger Delta region of Nigeria. The author’s extensive review of the literature and drawing conclusion from the empirical findings restate the neglect of the region and the consequences of pollution as a drawback to economic progress. The study further concludes that the greatest negative tendency associated with the exploration and exploitation of oil in this region is environmental degradation. However, a recent study by [20] suggests soil screening and massive clean-up funding to enhance contaminated land legislation. The efforts of government in the recently commissioned clean-up exercise of affected areas in the Niger Delta could not be ascertained but given the importance of oil exploration and exploitation to the Nigerian economy one would expect that this initiative will yield positive results.

[21], examined oil pollution and agricultural productivity in the Niger Delta of Nigeria, the study employed an empirical analysis derived from a unique estimable production function based on Ramon Lopez’s Cobb Douglas production function model. Findings established that increasing levels of oil spill and forest loss negatively affect agricultural productivity, while land, labour and capital positively improved agricultural productivity in the Niger Delta. In the same vein, [22] explore the environmental effects of petroleum activities and policies in Nigeria employing descriptive techniques to attain logical interpretations. Findings from this study revealed that the actions of oil companies operating in the Niger Delta have tremendous influence on the survival of ecosystems and biodiversity of the region. In a similar study, [23] investigates the consequences of oil spill on sea-food safety in coastal areas of Ibeno, Akwa Ibom State, observed the mean concentration of toxic petroleum hydrocarbons in the tissues of various fish species sample to be increasing as a result of oil spills. Investigating the impact of petroleum activities on various episodes of economic crisis in Nigeria, [11, 24] evaluates the historical pattern of oil spills using a descriptive technique to analyse data obtained from secondary sources and affirming that the transmogrification of the economy from agricultural-based to petroleum-based laid the foundation for the current economic crisis in Nigeria. Also, [25] while exploring the extent of environmental degradation in Niger Delta region and examining the efforts of oil companies in remediating the degraded farmlands in Niger Delta finds that oil pollution causes damage to human health, agricultural land and fish ponds as well as long-standing ecological malfunctioning.

[26] examining the effects of environmental degradation on human health in nine selected oil communities in Delta State, Nigeria using cluster and principal component analysis, observed that gas flaring has a statistically significant, but dangerous impact on human health in the affected areas giving the high temperature and emission to the atmosphere. Nonetheless, the problem of illegal bunkering and vandalizing petroleum pipelines contribute immensely to oil spillage and degradation of the environment. [27] observes that oftentimes illegal bunkering and petroleum pipeline vandalization results from destructive tendencies of restive youths, who were aggrieved by government neglect of oil producing communities and corruption of the ruling class in amassing wealth through collaborations with oil companies [28]. Unfortunately, these social vices perpetrated by the youths have a counter-effect in increasing the level of oil spills on the environment and the negative effect on water and land agricultural produce. Also, [29] analyse the influence of petroleum on Nigerian economy using secondary annual data from 2000 to 2009. The technique employed for the analysis include linear regression model and found that petroleum has substantial direct influence on the economy. Unfortunately, the mismanagement of the proceeds from petroleum exploration imbues Nigeria into the resource curse dilemma [28, 30]. Given the present circumstances in the Niger Delta and the need for improved economic activities for the population, it becomes very imperative for studies to explore the impact of environmental degradation on specific issues such as fish production to enable policy makers pin-point areas of concentration in the implementation of various policies for the economic development of the region.

Recent quantitative assessments of oil spills on fish production for other regions, especially in the Arctic, categorized fish in two distinct ways, [3] described the pelagic and demersal fish as varying with respect to their natural habitat and the impact of oil spills. The study contends that while the pelagic and fish eggs may come into direct contact with oil spill, the demersal fish does not easily come into contact with spilled oil, except it spills deep into the sea bed. However, prolonged spills may inhibit the survivability of the demersal fish to avoid spilled oil by swimming away from danger zone. The implication is that if a spill is allowed to spread, it permeates deep down to the sea bed over a long period, causing more harm to the environment, which in turn affects fish production. In the same vein, [1] assessing the impact of simulated oil spills on the Northeast Arctic Cod fishery, finds a spatial regeneration of fish population. In all simulations, the adult fish population remained at full reproductive potential with a reasonable number of juveniles swimming to replace the old fish population. Nevertheless, the variation in age of the fish determines the rate of survival following the impact of an oil spill. The study concludes that the reproductive health of the adult fish population is not affected in all simulations. However, the results provide the necessary insights to assist in the management of oil spills on fisheries.

Another very compelling study, [2] using data from the Northeast Arctic cod to estimate spatial variations in natural mortality segregates studies on the impact of oil spills on fish into retrospective and prospective studies. While the former investigates the impact of a spill, the later estimates the probable outcome of potential future oil spills. In this case, the prospective study finds that spatial variation in natural mortality can alter the impact of an oil-spill on fish. However, in this study we have employed the retrospective dimension to analyse the effects of oil spill on fish production in the Niger Delta. Here we strongly aver to the contrary on the assertion in [2] that scientific studies on the impact of oil spills on fish stocks tend to ignore the fact that spatial patterns of natural mortality may influence the magnitude of the impact over time. To this end, we apply parametric Cobb-Douglas production function on fish production and oil spill data, assuming that oil spills kill fish at the egg or larval stage and at maturity. The oil spill risk assessment is based on a probability of constant mortality rate for all fish categories, hence the spatial variability is difficult to estimate given the paucity of data.

## Stylized facts

### The extent of oil spillage

Oil spill incidents have occurred at different times along the Niger Delta area. From the records of the Department of Petroleum Resources (DPR) [31 – 32], within the period 1976–2015, a total no of 16,476 spills occurred at different occasions and a total quantity of approximately 3 million barrels spilled into the environment. Unfortunately, more than 70% was not recovered, 69% of these spills occurred off-shore, a quarter was in swamps and 6% spilled on land [33].

In [34] the record from the Nigerian National Petroleum Corporation (NNPC) indicates that the amount of crude oil spilled into the Niger Delta is estimated at 2,300 cubic metres, on average over 300 spills occurred every year from 1975 to 1995. In contrast, the World Bank provides figures [5 – 6] which estimates oil spill to the environment at almost ten times the NNPC figures arguing that the official figures ignore the so called “minor” spills. In the same vein, [8] categorically describes the largest individual spills to include the blowout of a Texaco offshore station in 1980, which dumped an estimated 400,000 barrels (64,000 m3) of crude oil into the Gulf of Guinea and Royal Dutch Shell, Forcado Terminal tank failure with an estimated spillage of 580,000 barrels (92,000 m3). [35] has modestly estimated the quantity of petroleum products and crude oil spilled in the Niger Delta through oil exploration activities as falling between 9 million and 13 million barrels.

## Methodology

### Source of data

This study employs time series data from 1981 to 2015 sourced from Central Bank of Nigeria Statistical Bulletin (2015), Department of Petroleum Resources (2016), and Food and Agricultural Organisation (2017) statistical bulletin. The data for fish production measured in tonnes and fishery loan and labour was obtained from Food and Agricultural Organization. The data for quantity of oil production and oil spill in barrels, was obtained from the Department of Petroleum Resources and Central Bank of Nigeria, Statistical Bulletin.

### Model specification

This study adopts a Cobb Douglas production model as specified by [21] which was employed to assess the influence of oil pollution on agricultural productivity in the Niger Delta of Nigeria.

The implicit model is stated thus;

$$FISP_{t}=f\left(OILS_{t},\;OILP_{t},\;FCAP_{t},\;FLAB_{t}\right)$$ (1)

Where;

FISP_t_: Fish production by captured fish measured in tones at period t.
OILS_t_: Quantity of oil spills in barrels at period t.
OILP_t_: Oil production in barrels at period t.
FCAP_t_: Fish capital proxy by number of fishery loan at period t
FLAB_t_: Fish labor captured by number of fishers at period t

The dependent variable is Fish production (FISP) measured in metric tons as the total fish production or output captured from inland and marine waters. Aquaculture value is excluded because this research is examining natural fish production and not agricultural sources. Also, the fishery statistical data presented excludes the production for marine mammals such as crocodiles, corals, sponges, pearls, shells and aquatic plants.

Independent variables are: Oil spills (OILS) measured in barrel during production, transportation and vandalization process officially recorded annually by oil companies in barrels. Oil production (OILP) is the value of total quantity of crude oil produced in Nigeria and measured in barrels annually as officially reported by the various oil companies, Fishery Labour (FLAB) is the total number of fishers involved in fish production, which include both artisanal and industrial sectors in fishing business and Fish capital (FCAP) is proxy by the value of fish production loan through Agricultural Credit Guarantee Scheme Funds.

Eq (1) can be rewritten explicitly as follows:

$$FISP_{t}=\text{A}\;OILS_{t}^{\beta 1}.OILP_{t}^{\beta 2}.FCAP_{t}^{\beta 3}.FLAB_{t}^{\beta 4}.\varepsilon_{t}$$ (2)

By adopting a double-log transformation of the model specified in Eq (2) through taking the natural logarithm of both sides of equation and assuming linearity among the variables. A is an efficiency parameter which accounts for the total factor productivity of the input and output variables in the model. In this case the model specification is transformed to a log-linear model for the purpose of estimation. The usefulness of this transformation includes minimization of the huge differences in the magnitude of different variables, thereby bringing out the coefficient of co-variation better and the explanation of the results in the form of elasticity with easily understandable interpretation devoid of complication from measurement unit.

$$LogFISP_{t}=\beta_{0}+\beta_{1}logOILS_{t}+\beta_{2}logOILP_{t}+\beta_{3}logFCAP_{t}+\beta_{4}logFLAB_{t}+\varepsilon_{t}$$ (3)

Where

Log: Natural log of the respective variables.
*ε*_t_: stochastic term (with the usual properties of zero and non-serial correlation)
*β*_0_: constant term;
*β*_1_, *β*_2_, *β*_3_, *β*_4_ are elasticities showing the degree of responsiveness of the dependent variable FISP to a proportional change in the independent variables; OILS, OILP, FCAP, FLAB

#### A priori expectation

*β*_1_ < 0, *β*_2_ < 0, *β*_3_ ≥ 0, *β*_4_ > 0

#### Estimation techniques

**Fig 1:**
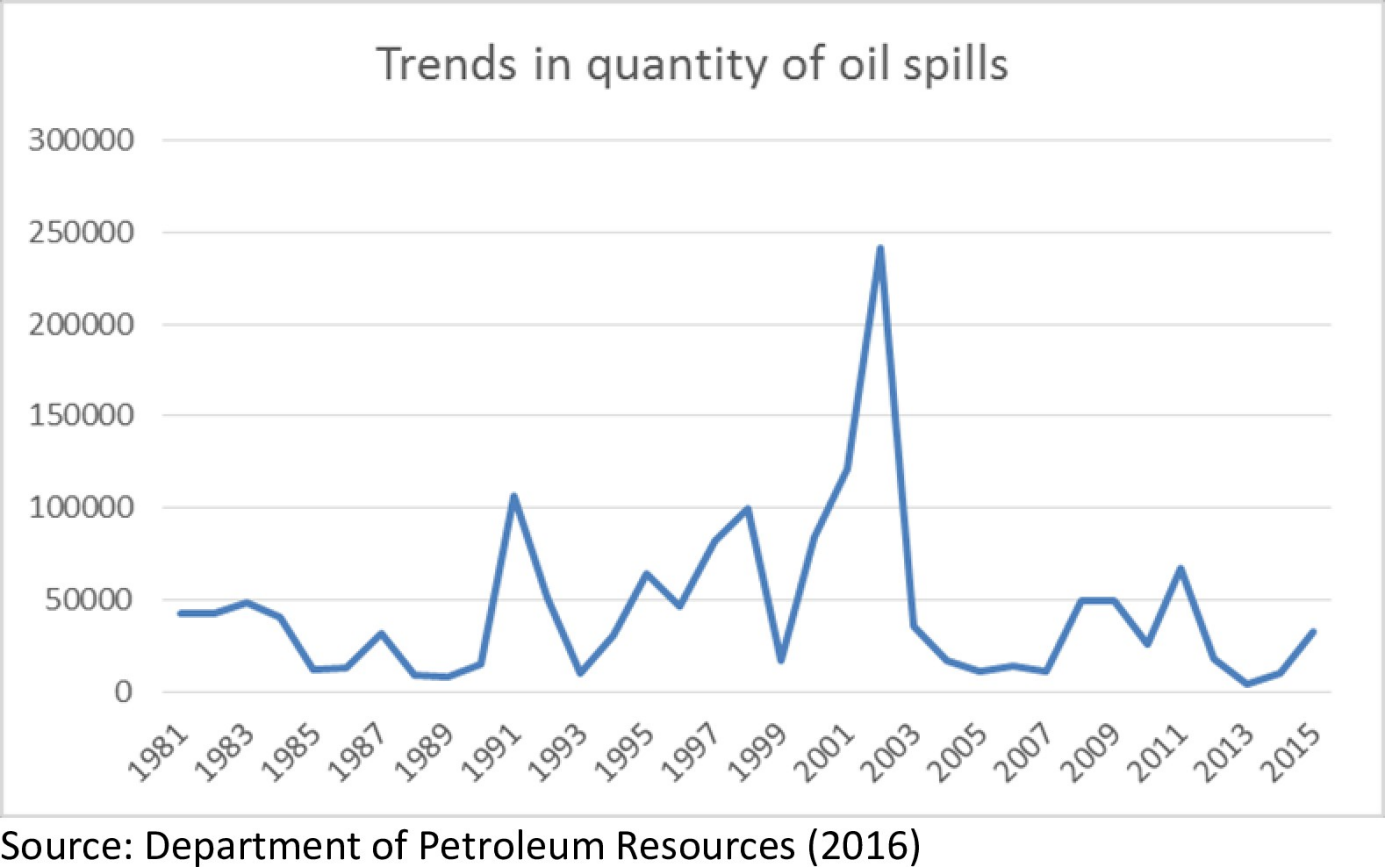
Graph showing the volume of oil spills in barrels 1981–2015.

**Fig 2:**
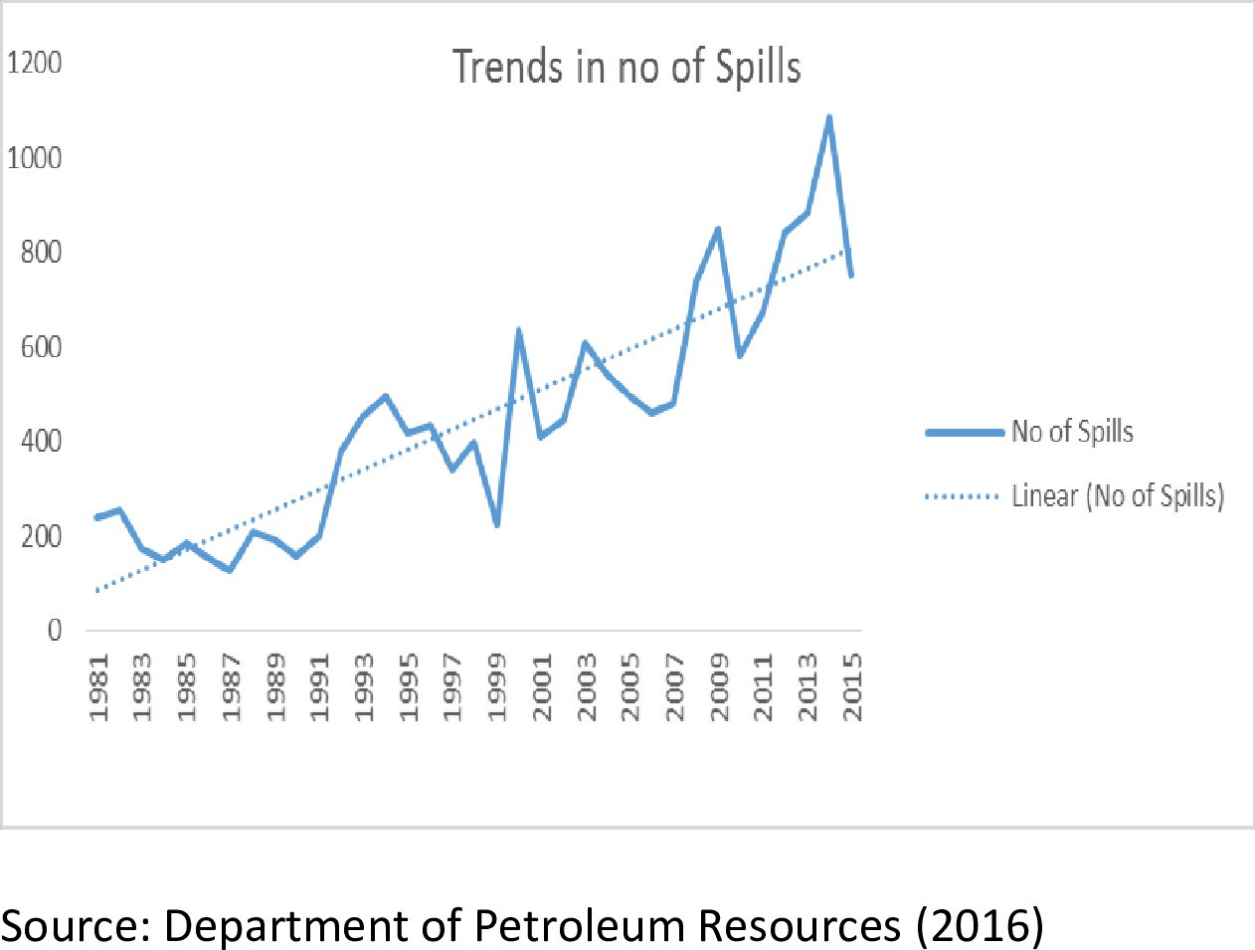
Trend of oil spills in barrels (1981–2015).

**Fig 3:**
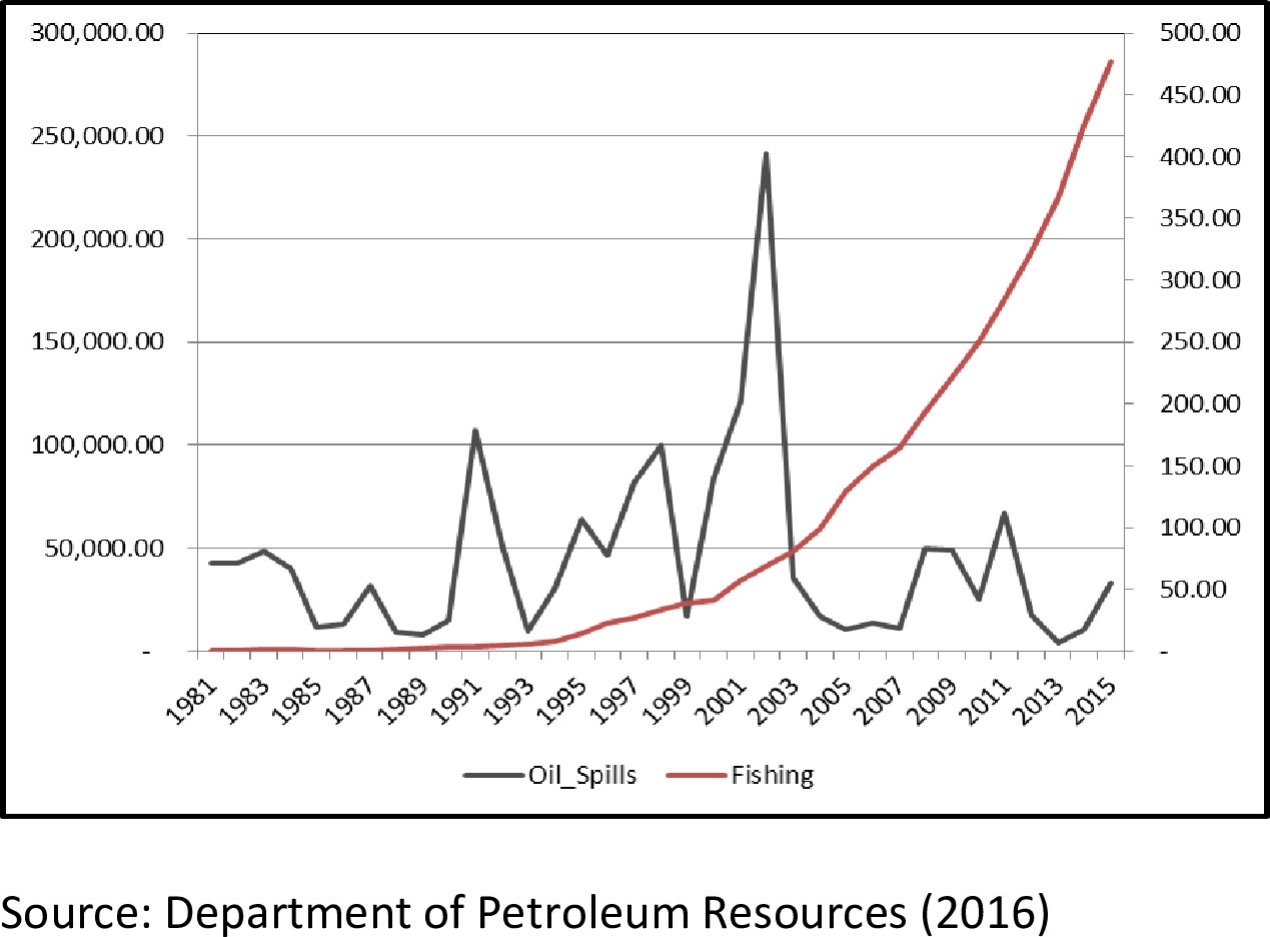
Graph of oil spills (Barrels) and fishing outputs (N’ billions) of Nigeria (1981–2015).

This study employs econometric technique to assess the relationship between oil spills and fish production among other associated variables. The descriptive method consists of trend graphs as shown in Figs 1, 2 and 3. The trends of oil spillage and fish production explains the behaviour of the variables from 1981 to 2015. The scope captures the dynamics of the explanatory variables and the effects on fish production in the Niger Delta region. The econometric methods adopted include Augmented Dickey Fuller Stationarity test, Johansen co-integration test, Fully Modified Ordinary Least Square (FMOLS) and Pairwise Granger Causality Test. This study employed the Augmented Dickey Fuller unit root to test for stationarity of variables because most time series are non-stationary at their levels, Co-integration is used to test for long run relationship between the dependent variable and the independent variables, Fully Modified Ordinary Least Squares is used to estimate the long run effect of the independent variables on the dependent variables after correcting for the endogeneity problem in the time series and Pairwise Granger Causality Test.

## Interpretation and discussion of results

### Interpretation of results

This section deals with the analysis and the interpretation of findings from the specified model based on a test of the research hypothesis. The parameter estimates are tested on econometric specifications. Inherently asymmetric data are normalized with logarithm. Stationarity tests of time series data are conducted to stabilize the mean, variance and autocorrelation properties that are presumed to be unstable over time. The cointegration and error correction specifications are obtained in order to elicit statistical relationships between variables, and a Granger causality test determines if there exists a causal link between oil spills and fish production and the direction of causation.

#### Stationarity test

The decision rule for the Augmented Dickey Fuller (ADF) Unit root test states that the PP Test statistic value must be greater than the Critical Value at 5% absolute terms for stationarity to be established at level and if otherwise, differencing occurs using the same decision rule [36]. Thus, the summary of results of the Augmented Dickey Fuller (ADF) unit root presented in Table 1 below shows that all the variables are stationary after first difference. This implies that the variables are all integrated at order one symbolized as **I (1)**.

**Table 1:** Results of Augmented Dickey Fuller (ADF) unit root test.

Variables	ADF Test Statistics		Critical Value		Order of integration	Remarks
	Level	1^ST^ diff	1%	5%		
LOGFISP	0.9628	-8.3643	-3.6463	-2.9540	I(1)	First Difference
LOGFLAB	-1.0359	-7.8305	-3.6395	-2.9511	I(1)	First Difference
LOGFLON	-0.5447	-9.3702	-3.6537	-2.9571	I(1)	First Difference
LOGOILP	-1.6021	-5.6437	-3.6394	-2.9511	I(1)	First Difference
LOGOILS	-0.3276	-6.2749	-2.6347	-1.9510	I(1)	First Difference

Source: Author’s computation (2017) from E-view (8.0)

#### Co-integration test

The co-integration test establishes whether a long-run equilibrium relationship exist among the variables of interest. Since the unit root test revealed that all the variables are integrated of order 1, Johansen Co-integration test can be applied.

#### Test of co-integration hypothesis:

H_0_: φ = 0 (No co-integrating equation)
H_1_: φ ≠ 0 (co-integrating equations)

The results of unrestricted trace co-integrating rank test suggest that the null hypothesis (H_0_) of no co-integrating equation is rejected and suggests the presence of one co-integrating equation at 5 percent significance level. Also, the unrestricted max-eigen co-integrating rank test rejects the null hypothesis (H_0_) of no co-integrating equation and suggests the presence of one co-integrating equation at 5 percent significance level (as shown in Table 2). Hence, we conclude that both unrestricted trace co-integrating rank test and unrestricted max-eigen co-integrating rank test confirmed the presence of co-integrating equation. Hence, there is a long run relationship between the dependent variable (LOGFISP) and the independent variables (LOGOILS, LOGOILP, LOGFCAP and LOGFLAB).

**Table 2:** Unrestricted co-integration rank test result.

No. of CE(s)	Eigen Value	Trace Statistics	0.05 Critical Value	Max-Eigen Statistics	0.05 Critical value
None	0.7183	85.2021	76.9728**	39.2774	34.8059**
1	0.4901	45.9246	54.0790	20.8779	28.5881
2	0.3570	25.0467	35.1928	13.6909	22.2996
3	0.2251	11.3558	20.2618	7.90561	15.8921
4	0.1053	3.45017	9.16455	3.45017	9.16455

Source: Author’s computation (2017) from E-view (8.0)

NOTE: (**) denotes rejection of hypothesis at 5% level of significance. Trace test and Max-eigenvalue test indicate 1 co-integrating equation each at the 0.05 level

#### Fully modified ordinary least square (FMOLS)

The long run adjustment dynamics can be usefully described by the Fully Modified Ordinary Least Square (FMOLS). FMOLS models are categories of multiple time series models that directly estimate the long run effect of the independent variables on the dependent variable after correcting for the endogeneity problem in the time series (Robin, 2008). FMOLS is also referred to as co-integrating equation model.

#### Goodness of fit

The goodness-of-fit is justified by the value of the coefficient of determination R squared. The adjusted R squared of 0.7013 indicate the explanatory variables in the model explains that 70 percent variations in fish production is jointly explained by number of fishers, credits to fishers, oil production and oil spills in Nigeria, while 30 percent of variation in the dependent variable are assumed to be due to error term or omitted variables. The F statistics confirmed that the model is statistically significant at 5 percent significant level (as shown in Table 3).

**Table 3:** Summary of FMOLS results.

**Dependent Variable:** **LOGFISP**	**Coefficient**	**Std. error**	**t-statistic**	**P-value**
LOGFLAB	0.7153	0.019609	39.47806	0.0000**
LOGFLOAN	-0.0051	0.004644	-1.090872	0.2846
LOGOILP	-0.4319	0.036292	-11.90307	0.0000**
LOGOILS	-0.0359	0.006375	-5.638374	0.0000**
Constant	12.4855	0.647376	19.28704	0.0000**
Adjusted $R^{2\;}$ = 0.70				

Source: Researcher’s computation (2017) from E-view (8.0

NOTE: (**) denotes rejection null hypothesis at 5% significance level

#### Statistical tests

The long run estimates presented in Table 3 reveal that number of fishers (FLAB), oil production (OILP) and oil spills (OILS) in Nigeria are statistically significant at 5 percent significance level. But, loan to fishers or fish production is statistically insignificant to explain changes in fish production at 5 percent significance level.

Specifically, a 1% increase in the number of fishers induces 0.72 percent rise in fish production in the long run given that other variables are held constant. However, 1 percent increase in oil production induces 0.43 percent decline in fish production and 1 percent increase in oil spills induces 0.04 percent fall in fish production in the long-run (see Table 3).

#### Pairwise granger causality test

[37] concept of causality occurs when time series $X_{t}$ and $Y_{t\;}$are co-integrated; a linear combination of $X_{t}$ and $Y_{t}$ must be stationary for further econometric tests to be carried out. Granger causality tests the difference between the two types of causation that exist between two variables (unidirectional and bidirectional causation). Unidirectional causality implies that if A Causes B, B cannot cause A, while bidirectional causality occurs if A Causes B then B causes A.

Using this concept, we fail to reject the null hypothesis that fish production does not Granger cause the number of times oil is spilled on the environment since its P-value (0.30) is greater than 0.05 significance level (see Table 4). However as shown in Table 4, we reject the null hypothesis in favour of the alternative hypothesis which states that the number of times oil is spilled on the environment affect the level of fish production; thus, we make a case of unidirectional relationship arguing that the number of times oil is spilled influences the level of fish production in the Niger Delta of Nigeria. Worthy of note is that causality in the Granger sense does not significantly imply correlation of oils spills and fish production in the Niger Delta. Nonetheless, we exercise caution in the fact that other seasonal factors may determine changes in the quantity of fish produced in the region. A statistical relationship of causation does not necessarily imply correlation, but in this case, we see a subjective pattern that warrants the conclusion drawn for this study.

**Table 4:** Pairwise granger causality tests.

Sample: 1981–2015			
Null Hypothesis:	Obs	F-Statistic	Prob.
LFISP does not Granger Cause LNO_SPILLS	34	5.14301	0.305
LNO_SPILL does not Granger Cause LFISP		4.10906	0.0513

Source: Author’s Compilation using EVIEWS 9.0

## Discussion of results

This study confirms the adverse effect of increase in oil spills on fish production in the Niger delta region of Nigeria. Oil spills are usually due to continuous incidence of vandalism and corrosion of oil pipelines, which destroy aquatic life and pollute the environment such that agricultural activities become impossible in the affected areas. The long-term effect of an oil spill incidence is usually associated with a reduction in crop yield and death of fish. This study corroborates the findings in [21] that oil spill is a major impediment to agricultural activities in the Niger Delta region of the country. In addition, it provides an empirical impetus for the findings in [38] for the management of petroleum hydrocarbon contaminated sites in Nigeria. Nevertheless, several studies have shown that the pollution caused by oil spillage does not end with the mopping up of the spilled oil in the land area or water [39]. It is now known that health risk is not averted by abstinence from fish killed by spilled oil. Some of the fishes and animals that escape instant death from pollution are known to have taken in some of the toxic substances, which in turn get into human beings that eat them. This will in turn cause infections on man coupled with other “side effects in form of genetic mutations” [40 – 41]. There is strong scientific proof that fish is a major indicator for environmental contamination, providing evidence for transmission of pollutants in marine ecosystems [33]

In fact, oil activity depresses fish production in the long run because of the unwholesome environmental degradation that accompany exploration of crude oil in the region. Oil driven environmental factors affecting fishing activities include gas flaring, oil well blowouts, and improper disposal of drilling mud, and pipeline leakages as observed in [42] and in [20], suggesting the prioritisation of sites for the clean-up exercise in the Niger Delta region, equally noting that high risk areas may not necessary imply the most contaminated zones, but based on the observed levels of hydrocarbon contamination and importance of the zone to the livelihood of the inhabitants.

Furthermore, this study finds that more labour involvement in fish production improves fish outputs in the region, exerting a positive and substantial influence on fish production. Sustainable improvement in agricultural sector requires skilled and able-bodied youths to engage in the agricultural process. This would drastically increase agricultural outputs in the country providing jobs for the unemployed youths and reducing incidence of restiveness in the region. However, credit to fish farmers through the Agricultural Credit Guarantee Scheme Funds (ACGSF) exert negligible, inverse and insignificant effect on fish outputs in the long run. This finding confirms the outcome of the studies by [43–46] that the Agricultural Credit Guarantee Scheme Fund (ACGSF) has no significant impact on agricultural production. This may be as a result of some challenges affecting the effectiveness of the scheme. Some of the challenges include a high rate of loan default by farmers; lack of full cooperation by participating banks.

This study also supports the assertion that the nature of operating equipment used by the oil companies, including pipeline vandalization by errant youths of the region are contributory factors to the number of oil spills on the environment, which constitute a setback to fish production and agricultural productivity resulting from the destruction of the environment. This result is in tandem with the observation in [10] on the socioeconomic consequences of oil spill on the environment, recommending an improvement in the infrastructure and equipment in order to prevent oil spills and the attendant youth restiveness resulting from deprivation.

## Conclusion

This study concludes that there is a trade-off between oil exploitation activities and fish production due to the effect of oil spills. We demonstrate that increasing levels of oil spillage and oil production negatively affects fish production in the Niger Delta region of Nigeria. However, changes in fish production may be stimulated by other seasonal and environmental factors not accounted for in our model of estimation. Frankly, incidence of oil spills among other environmental factors depress agricultural outputs particularly fishing. Also, agricultural interventions in Nigeria such as Agricultural Credit Guarantee Scheme Fund (ACGSF) failed to substantially improve fish production in Nigeria. Furthermore, labour input in fishing agricultural sub-sector can be employed towards improving productivity in the subsector. A major policy implication arising from the empirical evidence in this study is the need for an enhanced social protection policy for the inhabitants of the Niger Delta because providing credit for agricultural purposes may not yield significant results because of the short supply of arable land for cultivation and clean water for the survival of an aquatic ecosystem. In the same vein, since most of these riverine communities are traditionally into peasant fish production a destruction of the habitat completely dispossesses them of their productive capacity. This informs why there is a high loan default amongst loan recipients. The inhabitants of this region need to be educated on the process of commercial fishing that is required for the repayment of loans.

The Department of Petroleum Resources should enforce policies on pipeline life span duration, in order to reduce corrosion of pipelines. The adoption of the Special Partnership Framework (SPF) will help significantly in reducing oil-induced environmental diseconomies in the Niger Delta, including second-round consequences that undermine peace in the region, which adversely affects both oil and agricultural outputs. Improved pipeline quality and monitoring would also lessen the incidence of vandalism.

On the other hand, government should be prompt in the clean-up of the affected areas, by enacting and enforcing stringent environmental laws that will protect oil producing areas. Government should be able to identify natural resources (such as wetlands and coastal zones) in Nigeria and monetary investment in environmental protection of vulnerable areas should be seriously considered. There should be an operating standard for the examination of the existing water quality and monitoring, in addition to active monitoring and evaluation systems for water-related projects and services in the region.

Finally, we recommend the establishment of a framework for collaboration through training and financial support by government to strengthen environmental agencies and organizations in their role as watchdog for ensuring the exchange of information, especially for high risk oil extracting activities.

## Supporting information

S1 Dataset Research dataset. (XLSX)
